# Editorial: Unveiling the potential of microbiome in semi-wild and wildlife animals: exploring opportunities for disease mitigation and animal health across ecological zones

**DOI:** 10.3389/fmicb.2025.1623783

**Published:** 2025-06-06

**Authors:** Shuhao Bian, Shuyao Zhu, Hui Zhang, Yung-Fu Chang, Aoyun Li

**Affiliations:** ^1^College of Veterinary Medicine, Henan Agricultural University, Zhengzhou, China; ^2^College of Veterinary Medicine, South China Agricultural University, Guangzhou, China; ^3^Department of Population Medicine and Diagnostic Sciences, College of Veterinary Medicine, Cornell University, Ithaca, NY, United States

**Keywords:** microbiome, semi-wild, wildlife, disease, ecological zones

The microbiome is a complex community of microorganisms, including bacteria, fungi, viruses, and protozoa, that plays a crucial role in the animal health and survival (Alqahtani et al., 2024; Xie et al., 2024). Recently, the microbiome has attracted substantial interest because of its vital importance in maintaining the health and regulating the physiological processes of the host organism. Numerous investigations have indicated that the microbiome influences multiple physiological processes, including digestion, immune function, and defense against pathogens in both semi-wild and wild species (El-Sayed et al., 2021; Meng et al., 2024). Wild herbivores possess a diet rich in cellulose. However, they lack the ability to produce enzymes necessary for its breakdown. Conversely, certain bacteria, fungi, and protozoa present in the gut microbiome can secrete cellulase, an enzyme that degrades cellulose into absorbable sugars (Weimer, 2022). Studies have demonstrated that fiber-decomposing bacteria in the rumen can adhere to the surfaces of plant fibers, secreting cellulase to gradually degrade cellulose into monosaccharides. These monosaccharides are then further fermented to produce volatile fatty acids, which provide energy for animals (Matthews et al., 2019; Chen et al., 2022). The gut microbiome is capable of producing short-chain fatty acids (SCFAs) during the fermentation of carbohydrates, such as dietary fiber and oligosaccharides (Parada Venegas et al., 2019; Dupraz et al., 2021). SCFAs significantly influence the immune system. Over the past decade, SCFAs have garnered considerable attention due to their effects on host immune responses. Research has indicated that SCFAs regulate epithelial barrier function and mucosal as well as systemic immunity via evolutionarily conserved mechanisms that involve G protein-coupled receptor signaling and histone deacetylase activity (Mann et al., 2024). Yang et al. (2020) also showed that supplementation with SCFAs can enhance the production of IL-22, thereby offering protection to the intestine against inflammation. Interestingly, there are also certain microbial groups on the surface of the skin and mucosa, which build a complex ecological network with each other. Different species of bacteria cooperate with each other to form a dynamic balance. Once foreign pathogens invade, the group defense mechanism will be triggered. Dokoshi et al. (2024) found that in a mouse model, skin damage can establish a connection from the skin to the intestine, which will cause changes in the composition and function of the gut microbiome and also disrupt the balance of intestinal immunity. Consequently, maintaining the microbiome stable is necessary for the host's health. With the increasing significance of the microbiome in animal health, increasing research is devoted to exploring the potential links between the microbiome and the host. This unique electronic collection encompasses several articles that address these multifaceted aspects.

*Rhinopithecus brelichi*, a rare species endemic to the Fanjingshan National Nature Reserve in northeastern Guizhou, China, plays a crucial role in the ecosystem. Owing to fragmented habitats, a prolonged reproductive process, and limited genetic variation, the current population size of this species is around 400 individuals. Consequently, it has been categorized as a critically endangered species on the IUCN Red List of Threatened Species. To protect this endangered species, wildlife breeding centers and zoos have implemented artificial breeding strategies. However, parasitic infections significantly impact the health of *Rhinopithecus brelichi*, jeopardizing the survival and development of artificial breeding populations and presenting a challenge for the conservation of this species. Despite the considerable influence of parasitic infections on *Rhinopithecus brelichi*, relatively few studies have investigated the response mechanisms of their gut microbiome to anthelmintic treatments. Qin et al. conducted a comprehensive study on *Rhinopithecus brelichi* before and after albendazole treatment, revealing a close association between anthelmintic treatment and alterations in gut microbiome and metabolites. More precisely, the administration of anthelmintics resulted in a marked decline in the number of worm eggs present in each gram of feces. The results of 16S rRNA gene sequencing indicated a marked increase in the richness and diversity of the gut microbiome following deworming. Concurrently, deworming procedure also altered the composition of the microbiome. These significant changes in gut microbiome and metabolites may be critical factors influencing the health status of *Rhinopithecus brelichi* and are likely closely related to parasitic infections and physiological changes following deworming treatments.

Yaks, a native species specific to the Qinghai - Tibet Plateau, display excellent adaptability to the hypoxic conditions that are characteristic of this region. Moreover, yaks function as a fundamental form of conveyance for the inhabitants of the Qinghai - Tibet Plateau and offer key resources like milk, meat items, and hides. However, the stunted growth of yaks presents a major obstacle to the yak breeding industry in high-altitude regions, seriously endangering the economic feasibility of yak husbandry and the sustainable development of local animal husbandry. Despite its considerable impact, relatively few studies have investigated the potential relationship between yak growth retardation and the yak microbiome. Wang H. et al. conducted a comprehensive study comparing low- and high-weight yak calves alongside their respective mothers, revealing that variations in yak weight are closely linked to alterations in the microbiome composition of both mother yaks and calves. Specifically, weight differences influence multiple taxonomic levels of the microbiota. In female yaks, nine genera, including *Avispirillum, Feneckera*, and CAG1031, exhibited significant differences between the higher weight and lower weight groups. In yak calves, high-weight yaks demonstrated striking contrasts in one phylum and six genera, such as CAG-485 and CAG-83, when compared to low-weight yaks. Notably, the ratio of *Firmicutes* to *Bacteroidetes*, an important indicator related to metabolism, also exhibited changes. These considerable shifts in the composition along with abundance of the gut microbiome may be critical factors influencing the growth and health status of yaks, and are likely to be closely associated with the phenomenon of growth retardation in these animals.

Recent advancements in microbiome research technology and a growing awareness of the significance of microbial communities in host health have catalyzed a surge in the study of animal skin fungal communities. This has greatly enhanced our understanding of the intricate relationships among fungi, hosts, and their environments. However, many studies on bat skin fungal communities still lack a comprehensive understanding of the ecological processes driving their assembly, which presents challenges for effectively protecting bat populations and understanding the interactions between pathogens and fungi. Additionally, the skin fungal communities of bats are closely linked to their health. Any disruption to these communities may adversely affect their survival and reproduction, posing potential risks to bat biodiversity. The skin serves as the primary defense barrier for bats against external environmental factors, indicating that both the skin and its associated fungal communities are inevitably influenced by various elements. Wang D. et al. discovered notable disparities in the composition along with diversity of skin fungal communities across various bat species in northern China. These differences were influenced by factors such as temperature, sampling location, and body mass index. Additionally, this research revealed that deterministic mechanisms, especially homogeneous selection, have a pivotal influence on the formation of skin fungal communities. Understanding this is essential for grasping how these communities adapt to environmental fluctuations. However, the diversity along with composition of bat skin fungal communities show notable differences compared to the environmental fungal reservoir. It implies that bats deliberately choose certain fungi from the environment to colonize their skin, and this process is governed by multiple ecological elements.
